# Semi-Annual Meeting of the Missouri Association

**Published:** 1887-01

**Authors:** 


### SEMI-ANNUAL MEETING OF THE MISSOURI ASSOCIATION.

The semi-annual meeting of the Missouri State Association of Veterinary Science and Comparative Medicine was held in December, at Dr. James', 1029 Leffingwell avenue. The Secretary read a paper by T. A. Edwards, M. D., of Marshall, Mo., on "Professional Worth." The doctor made one novel statement in his essay, that Michael Servetus was burned by Calvin because of his teaching the circulation of the blood.

Theologians usually say it was because Servetus was a kind of Unitarian. He went on to say that the physician, even more than the clergyman, must be a man of professional worth, a man of real unselfishness. He held that as veterinarians rightly conducted themselves they will earn respect. He was very severe on people who belittle the true veterinary profession. The paper was decidedly witty and sarcastic in parts.

Speaking of the paper, Dr. Paquin told of the chair of comparative pathology at Columbia. He showed the necessity of that study for the ordinary practitioner. Three men had died of glanders during the last year, and no physician was able to diagnose their cases. The fact was brought out that the quarantine law was excellent and very strict, if enforced.

Veterinary Surgeon James described a case of pannus in a horse's eye, which had come under his observation. He treated it with mucous from a pink-eye patient, then with nitrate of silver, and was thoroughly successful. The disease he had seen very frequently in dogs.

After this the President called attention to the fact that the sanitary law of the State provided only one veterinary surgeon for the whole State, and provided no assistants at any time. Besides, no provision was made for the study of special diseases, such as hog cholera. Dr. Tiffany described the provisions of Illinois law, where the practical veterinarians over the State were empowered as assistants to the Live Stock Commission. They were paid per diem only, and expenses.

Dr. Paquin was ordered to consult with Mr. Proctor, author of the law, on the subject.

The doctor gave an account of his visit to Europe, principally to study hog cholera. Dr. Salmon held that the French and American diseases were different. Externally the lesions were the same. Dr. Paquin was almost certain that the same germ caused each disease. Hog cholera was not vaccinated against with success, only for certain breeds. They inoculate through rabbits. The operation was a particularly delicate one. The doctor discussed it and kindred subjects at considerable length.

